# Neurological Severity and Systemic Biochemical Markers in Relation to Transition to Oral Feeding in Chronic Post-Stroke Dysphagia

**DOI:** 10.3390/jcm15176834

**Published:** 2026-09-03

**Authors:** Timur Orhanoglu, Merve Savaş, Senanur Kahraman Beğen, Abdulhalim Senyigit, Hafize Uzun

**Affiliations:** 1 Department of Internal Medicine, Faculty of Medicine, Istanbul Atlas University, 34403 Istanbul, Türkiye; abdulhalim.senyigit@atlas.edu.tr; 2 Department of Speech and Language Therapy, Faculty of Health Sciences, Istanbul Atlas University, 34403 Istanbul, Türkiye; merve.savas@atlas.edu.tr (M.S.); senanur.kahraman@atlas.edu.tr (S.K.B.); 3 Department of Medical Biochemistry, Faculty of Medicine, Istanbul Atlas University, 34403 Istanbul, Türkiye; hafize.uzun@atlas.edu.tr

**Keywords:** stroke, dysphagia, oral feeding, aspiration, Functional Oral Intake Scale, Penetration–Aspiration Scale, vitamin D, vitamin B12, biomarkers, rehabilitation

## Abstract

**Background:** Post-stroke dysphagia is associated with aspiration pneumonia, malnutrition, and poor functional outcomes. Although systemic biochemical abnormalities are common after stroke, their contribution to swallowing recovery and transition to oral feeding remains unclear. This study investigated the association of systemic biochemical markers with oral feeding transition and swallowing-related functional improvement in patients with chronic post-stroke dysphagia. **Methods:** This retrospective single-center study included 89 patients with chronic post-stroke dysphagia who received enteral nutrition between February 2023 and December 2025. Biochemical parameters and swallowing outcomes, assessed using the Functional Oral Intake Scale (FOIS) and Penetration–Aspiration Scale (PAS), were retrospectively analyzed. Changes between baseline (T0) and follow-up (T1) were calculated (Δ = T1 − T0). Multivariable logistic and linear regression analyses were performed to identify independent predictors of transition to oral feeding and improvement in aspiration severity. **Results:** Of the 89 patients, 52 (58.4%) achieved oral feeding. Significant improvements in swallowing function were observed following rehabilitation, as reflected by increased FOIS scores and decreased PAS-soft and PAS-liquid scores (all *p* < 0.001), together with reductions in CRP (C-reactive protein), D-dimer, white blood cell count, and neutrophil count (all *p* < 0.05). Patients who achieved oral feeding demonstrated greater improvements in PAS scores (both *p* < 0.001) and a divergent change in vitamin B12 levels (a decrease among those who transitioned versus an increase among those who remained enterally fed) (*p* = 0.049). In exploratory multivariable analyses, higher NIHSS (National Institutes of Health Stroke Scale) scores and lower baseline 25-hydroxyvitamin D levels were associated with a lower likelihood of transition to oral feeding (NIHSS OR = 0.78, *p* = 0.031; vitamin D OR (Odds ratio) = 1.12, *p* = 0.001), whereas baseline PAS-liquid score was not an independent predictor of transition. In the linear regression analysis of aspiration severity, baseline PAS-liquid score was the strongest predictor of ΔPAS-liquid (B = −0.527, *p* = 0.001), while lower baseline 25-hydroxyvitamin D was associated with less improvement (B = −0.021, *p* = 0.047). Given the limited sample size and the absence of correction for multiple comparisons, the biochemical associations should be interpreted as exploratory. **Conclusions:** In patients with chronic post-stroke dysphagia, transition to oral feeding was primarily associated with neurological severity, whereas improvement in aspiration severity was most strongly related to baseline aspiration burden. Lower baseline 25-hydroxyvitamin D showed exploratory associations with both outcomes. Because 25-hydroxyvitamin D did not change significantly during follow-up and the models were underpowered, this association should be regarded as hypothesis-generating rather than clinically established. These biochemical findings require confirmation in larger prospective studies.

## 1. Introduction

Stroke frequently impairs swallowing physiology, resulting in dysphagia and increasing the risk of serious pulmonary complications, particularly aspiration pneumonia. Systematic reviews have demonstrated a high prevalence of post-stroke dysphagia and a markedly increased risk of pneumonia in patients with aspiration [1]. Beyond respiratory complications, dysphagia substantially affects nutritional status. Malnutrition is common after stroke, particularly when biochemical parameters are included in nutritional assessments, and is independently associated with increased morbidity, mortality, and prolonged hospitalization [2,3]. Moreover, impaired nutritional status has been shown to limit functional recovery and adversely affect rehabilitation outcomes [4].

These findings suggest that recovery of swallowing function depends not only on local neurological mechanisms but also on the patient’s systemic biological condition. Peripheral factors, including neuromuscular integrity, myelin maintenance, and immunometabolic regulation, contribute to functional recovery. Accordingly, vitamin D and vitamin B12 have been investigated in relation to functional recovery and neurological integrity [5,6]. Likewise, D-dimer, reflecting thromboinflammatory burden, has been linked to disease severity and poor functional outcomes after stroke [7,8].

Systemic vulnerability can also be evaluated through electrolyte balance and renal and hepatic function. Renal dysfunction and poor nutritional status are associated with poorer outcomes after stroke [3,9]. In line with this concept, biological and nutritional status have been reported to influence functional gains and discharge outcomes during rehabilitation [4,10].

Recent evidence further supports the prognostic significance of biochemical markers in stroke. The aspartate aminotransferase/alanine aminotransferase (AST/ALT; De Ritis) ratio and the aspartate aminotransferase/alkaline phosphatase (AST/ALP) ratio independently predicted 3-month functional outcomes and mortality in acute ischemic stroke [11]. Similarly, elevated serum γ-glutamyl transferase (GGT) levels have been shown to independently predict one-year all-cause and cardiovascular mortality [12]. These findings suggest that liver-derived biochemical markers and related ratios may reflect the systemic biological burden beyond neurological injury.

Functional recovery in post-stroke dysphagia is most clearly reflected by improvements in oral intake and swallowing safety. The Functional Oral Intake Scale (FOIS) is widely used to classify oral feeding status, whereas the Penetration–Aspiration Scale (PAS) objectively grades the severity of airway invasion during videofluoroscopic swallowing studies [13,14]. The videofluoroscopic swallowing studies were performed using the standard institutional protocol of İstanbul Atlas University Hospital and were interpreted by two speech and language therapists, each with more than five years of experience in dysphagia assessment, who scored the examinations by consensus. All bolus volumes and consistencies specified in the protocol were evaluated. Because the studies were rated by consensus rather than independently, formal intra- and inter-rater reliability (e.g., intraclass correlation coefficient) was not calculated. Increases in FOIS scores indicate progression from enteral to oral feeding, whereas decreases in PAS scores reflect improved swallowing safety and reduced aspiration risk.

The primary aim of this study was to investigate the independent and relative value of systemic biochemical parameters in predicting the transition from enteral to oral feeding in patients with post-stroke dysphagia. We also evaluated whether these biomarkers were associated with changes in FOIS and PAS scores, which reflect improvements in swallowing function and safety. Because chronic post-stroke dysphagia is determined primarily by the site and extent of the neurological lesion and by bulbar motor control, systemic biochemical status is best regarded as a metabolic background for neuroplasticity that operates secondary to the primary neurological injury. In addition, swallowing recovery depends not only on airway protection but also on swallowing efficiency and pharyngeal clearance; because the Functional Oral Intake Scale and the Penetration–Aspiration Scale capture feeding status and airway safety rather than these physiological dimensions, the present outcomes should be interpreted accordingly. We hypothesized that biochemical markers not only represent general systemic status but also contribute to the prediction of functional outcomes during dysphagia rehabilitation. The primary hypothesis was that systemic biochemical parameters, including creatinine, urea, sodium, potassium, vitamin B12, 25-hydroxyvitamin D, ferritin, D-dimer, albumin, total protein, AST, ALT, GGT, and the AST/ALT (De Ritis) ratio, would independently predict the transition from enteral to oral feeding in patients with post-stroke dysphagia. The secondary hypothesis was that these biochemical parameters would also be independently associated with changes in swallowing-related clinical outcomes, as reflected by changes in the Functional Oral Intake Scale (FOIS) and Penetration–Aspiration Scale (PAS) scores.

## 2. Materials and Methods

This retrospective study was approved by the Non-Interventional Scientific Research Ethics Committee of Istanbul Atlas University (Date: 30 January 2026; Approval No. E-22686390-050.99-90336). All procedures were conducted in accordance with the principles of the Declaration of Helsinki. The requirement for informed consent was waived due to the retrospective nature of the study and the use of anonymized data.

### 2.1. Study Design

This retrospective, observational, analytical, single-center study was conducted to investigate the associations of systemic biochemical markers with the transition from enteral to oral feeding and swallowing function in patients with post-stroke dysphagia. The study was performed at Istanbul Atlas University Hospital, and medical records were reviewed retrospectively. Clinical, biochemical, and swallowing assessment data of eligible patients followed between February 2023 and December 2025 were analyzed.

### 2.2. Study Setting and Participants

The study population consisted of patients diagnosed with post-stroke dysphagia who received enteral nutrition during hospitalization at Istanbul Atlas University Hospital within the study period. A consecutive sampling approach was used, and all eligible patients were included.

A total of 89 patients were enrolled in the final analysis. During follow-up, 52 patients transitioned to oral feeding, whereas 37 remained dependent on enteral nutrition.

### 2.3. Inclusion and Exclusion Criteria

Patients were eligible if they had (i) clinically and/or radiologically confirmed ischemic stroke, (ii) a diagnosis of dysphagia, (iii) received enteral nutritional support via a nasogastric tube or percutaneous endoscopic gastrostomy during hospitalization, and (iv) available biochemical and swallowing assessment records. In addition, patients were required to have sufficient auditory comprehension and cooperation to participate in bedside and/or videofluoroscopic swallowing assessments.

Exclusion criteria included severe cognitive impairment or poor cooperation precluding reliable swallowing assessment, pre-existing advanced neuromuscular diseases, head and neck malignancies directly affecting swallowing function, and critical missing data.

### 2.4. Timing of Assessments

Assessments were performed according to routine clinical dysphagia management during the same hospitalization episode. Baseline (T0) corresponded to the initial swallowing evaluation performed during the enteral feeding period, whereas follow-up (T1) represented reassessment after completion of structured dysphagia rehabilitation under standard institutional care. Dysphagia rehabilitation was provided for approximately 6 weeks, with three sessions per week, and each session lasted approximately 30 min. The sessions were delivered by speech and language therapists experienced in dysphagia management. Conventional dysphagia rehabilitation methods were used, including swallowing maneuvers and oral sensory-motor stimulation, according to individual clinical needs. Neuromuscular electrical stimulation was not used. Transition to oral feeding was initiated following a formal clinical board decision based on the patient’s swallowing status and overall clinical condition. The duration of enteral nutrition before the baseline assessment varied among patients and could not be standardized because of the retrospective nature of the study.

FOIS and PAS scores were retrospectively extracted from clinical and videofluoroscopic swallowing study (VFSS) reports at both time points. Laboratory values were obtained from routine blood tests performed during the corresponding clinical periods to ensure temporal alignment between biochemical and swallowing evaluations. Longitudinal changes were calculated as Δ = T1 − T0 for all clinical and laboratory variables.

### 2.5. Definition of Transition to Oral Feeding

Transition to oral feeding was defined as achieving functional oral intake without tube dependency at follow-up assessment, corresponding to a FOIS score of ≥4 at T1. Within the FOIS framework, scores of 1–3 indicate varying degrees of tube dependence, whereas scores ≥ 4 indicate total oral intake without reliance on enteral nutrition, although dietary modifications may still be required [14]. Therefore, FOIS ≥ 4 was considered to represent clinically meaningful discontinuation of tube feeding and restoration of oral nutritional autonomy.

### 2.6. Data Collection

Data were retrospectively collected from electronic medical records and patient files. Biochemical data were obtained from laboratory records, whereas swallowing-related information was extracted from bedside evaluations, VFSS reports, and clinical follow-up notes. All data were anonymized and coded before analysis. Only patients with matched biochemical and swallowing assessment data were included.

### 2.7. Clinical Assessment Tools

#### 2.7.1. Stroke Severity

Baseline neurological severity was assessed using the National Institutes of Health Stroke Scale (NIHSS), a standardized 15-item neurological examination evaluating consciousness, motor function, sensation, language, speech, and neglect. Scores range from 0 to 42, with higher scores indicating greater neurological impairment [15]. In the present study, NIHSS was included as a potential confounding variable in multivariable analyses.

#### 2.7.2. Aspiration Severity

Aspiration severity was evaluated using the Penetration–Aspiration Scale (PAS), an eight-point ordinal scale that grades the degree of airway invasion during swallowing, with higher scores indicating more severe penetration or aspiration [13]. PAS scores for liquid and semisolid consistencies available in clinical records were analyzed.

#### 2.7.3. Functional Oral Intake

Functional oral intake was assessed using the Functional Oral Intake Scale (FOIS), a seven-level scale that classifies oral intake and tube dependency. Lower scores indicate tube dependence and restricted oral intake, whereas higher scores indicate more independent oral feeding [14]. FOIS has previously been shown to correlate with feeding method, dysphagia severity, and functional outcomes in patients with post-stroke dysphagia [14].

### 2.8. Biochemical Parameters

The following biochemical parameters were evaluated: creatinine (mg/dL), urea (mg/dL), alanine aminotransferase (ALT, U/L), aspartate aminotransferase (AST, U/L), sodium (mmol/L), potassium (mmol/L), vitamin B12 (pg/mL), 25-hydroxyvitamin D (ng/mL), ferritin (ng/mL), D-dimer (mg/L), albumin (g/dL), total protein (g/dL), gamma-glutamyl transferase (GGT, U/L), C-reactive protein (mg/L), procalcitonin (ng/mL), a complete blood count (white blood cell, neutrophil, lymphocyte, monocyte, eosinophil, and basophil counts), hemoglobin, hematocrit, platelet count, and the AST/ALT (De Ritis) ratio (Architect i2000, Abbott Laboratories, Abbott Park, IL, USA). These biomarkers were selected to represent systemic inflammation, nutritional status, thromboinflammatory burden, electrolyte balance, and organ function.

### 2.9. Outcome Measures

The primary outcome was transition to oral feeding, coded as a binary variable (0 = no, 1 = yes). Secondary outcomes included changes in swallowing-related clinical parameters, namely FOIS and PAS scores. Pre-rehabilitation assessments were defined as T0 and post-rehabilitation assessments as T1, and change scores were calculated using the formula Δ = T1 − T0. Accordingly, ΔFOIS and ΔPAS were analyzed as secondary outcome measures.

### 2.10. Statistical Analysis

Statistical analyses were performed using IBM SPSS Statistics for Windows, Version 26.0 (IBM Corp., Armonk, NY, USA). Continuous variables are presented as mean ± standard deviation (SD), median, minimum–maximum values, and 95% confidence intervals (CIs), as appropriate. Normality was assessed using histograms, Q–Q plots, skewness and kurtosis coefficients, and the Shapiro–Wilk test. Non-parametric methods were used when the assumption of normality was violated. Pre-rehabilitation measurements were defined as T0 and post-rehabilitation measurements as T1. Longitudinal changes were calculated as Δ = T1 − T0 for all clinical and laboratory parameters. The analyses were conducted in four stages. First, changes in laboratory parameters and swallowing-related clinical measures between T0 and T1 were evaluated using the Wilcoxon signed-rank test. Second, laboratory parameters and change scores were compared between patients who achieved oral feeding and those who remained enterally fed using the Mann–Whitney U test. Categorical variables were compared using the chi-squared test. Third, exploratory multivariable binary logistic regression models were initially constructed separately for each biochemical parameter to evaluate its association with transition to oral feeding after adjustment for age, sex, baseline NIHSS score, baseline PAS-liquid score, and stroke localization. As a secondary exploratory analysis, biomarkers showing statistical signals in these adjusted models were examined in greater detail. Because the vitamin D-focused logistic regression model showed quasi-complete separation and the number of events per parameter was limited, the model was re-estimated using Firth’s penalized-likelihood logistic regression to reduce small-sample bias. These penalized models were fitted in Python (version 3.11, NumPy) following Firth [16] and Heinze and Schemper [17], and multicollinearity among the model variables was acceptable (all variance inflation factors ≤ 1.42). The vitamin D-focused models included baseline 25-hydroxyvitamin D, Δ25-hydroxyvitamin D, age, sex, baseline NIHSS score, baseline PAS-liquid score, and stroke localization (middle cerebral artery as reference). Results are reported as odds ratios (ORs) with 95% confidence intervals and *p*-values. Model performance was summarized using Nagelkerke R^2^, the area under the receiver operating characteristic curve (AUC), and overall classification accuracy. Internal validation was performed using 2000 bootstrap resamples, and an optimism-corrected AUC was calculated. As a post hoc exploratory step, albumin, vitamin B12, D-dimer, and 25-hydroxyvitamin D were examined further based on the statistical patterns observed in the initial adjusted analyses and their clinical interpretability. These secondary analyses were considered hypothesis-generating. Fourth, additional analyses were performed for baseline 25-hydroxyvitamin D status. Baseline vitamin D levels were categorized according to the Endocrine Society classification as deficient (<20 ng/mL), insufficient (20–29 ng/mL), and sufficient (≥30 ng/mL) [18]. Continuous variables were compared using the Kruskal–Wallis test, whereas categorical variables were analyzed using the chi-squared test. To evaluate the effect of baseline vitamin D status on changes in swallowing function, multivariable linear regression models were constructed using ΔFOIS, ΔPAS-liquid, and ΔPAS-soft scores as dependent variables. These models were adjusted for age, sex, NIHSS score, and the corresponding baseline assessment score. Results are reported as regression coefficients (B), 95% confidence intervals, and *p*-values. The discriminatory performance of baseline 25-hydroxyvitamin D levels for predicting transition to oral feeding was further evaluated using receiver operating characteristic (ROC) analysis. The area under the curve (AUC) and its 95% confidence interval were calculated, and the optimal cutoff value was determined using the Youden index. Given the limited sample size, separate multivariable models were preferred to preserve model stability and reduce the risk of overfitting. Because multiple biochemical parameters were tested, no formal adjustment for multiple comparisons was applied. Therefore, all biomarker analyses were considered exploratory and hypothesis-generating rather than confirmatory. For change scores mathematically coupled to baseline (e.g., ΔPAS-liquid), an analysis of covariance with the post-treatment score as the dependent variable and the baseline score as a covariate was used as a sensitivity analysis. Statistical significance was defined as a two-sided *p*-value < 0.05. Owing to the retrospective design, a small number of laboratory values were missing; all analyses were performed using the available data for each parameter (pairwise-complete), so the effective number of patients varies slightly across parameters.

## 3. Results

A total of 89 patients with chronic post-stroke dysphagia were included. The mean age was 57.5 ± 22.1 years, and the mean time since stroke was 38.5 ± 7.8 weeks. Of the patients, 48 were female, and 41 were male. During follow-up, 52 patients achieved transition to oral feeding, whereas 37 remained dependent on enteral nutrition. The most common stroke localization was the middle cerebral artery territory. The demographic and clinical characteristics of the study population are presented in Table 1.

Following dysphagia rehabilitation, significant improvements were observed in both inflammatory markers and swallowing-related clinical outcomes (Table 2). CRP and D-dimer levels decreased significantly after therapy (both *p* < 0.001). Significant changes were also observed in WBC count, neutrophil count, lymphocyte percentage, monocyte count, basophil percentage, and sodium levels.

Regarding swallowing outcomes, FOIS scores increased significantly after therapy (*p* < 0.001), whereas PAS-soft and PAS-liquid scores decreased significantly (both *p* < 0.001), indicating improved oral intake and reduced aspiration severity. Changes in laboratory and clinical parameters were compared between patients who transitioned to oral feeding and those who did not (Table 3). Patients who achieved oral feeding showed significantly greater reductions in PAS-soft and PAS-liquid scores compared with those who remained enterally fed (both *p* < 0.001). No significant difference was observed in ΔFOIS or most laboratory parameters. The change in vitamin B12 differed significantly between groups (*p* = 0.049), with a decrease among patients who transitioned to oral feeding (−52.06 ± 275.41 pg/mL) and an increase among those who remained enterally fed (36.74 ± 162.47 pg/mL).

In the vitamin D-focused Firth penalized logistic regression model evaluating transition to oral feeding, the model showed a Nagelkerke R^2^ of 0.51 and an area under the ROC curve of 0.88. In an exploratory comparison, adding baseline 25-hydroxyvitamin D to a model containing only the clinical variables (NIHSS score, age, sex, baseline PAS-liquid score, and stroke localization) improved discrimination from an AUC of 0.77 to 0.86 (penalized likelihood-ratio *p* < 0.001); given the small sample, this incremental finding is hypothesis-generating, and most other biomarkers did not add discriminative value. Following 2000-resample bootstrap internal validation, the optimism-corrected AUC was 0.81, and the overall classification accuracy was 84.4% (Table 4). Higher NIHSS scores and lower baseline 25-hydroxyvitamin D levels were independently associated with a lower probability of transition to oral feeding (NIHSS OR = 0.78, 95% CI: 0.62–0.98, *p* = 0.031; vitamin D OR = 1.12, 95% CI: 1.05–1.20, *p* = 0.001). In contrast, baseline PAS-liquid score was not an independent predictor of transition (OR = 1.08, 95% CI: 0.47–2.48, *p* = 0.863).

A multivariable linear regression model was used to identify independent determinants of improvement in aspiration severity, using ΔPAS-liquid as the dependent variable (Table 5). The model was statistically significant (*p* < 0.001) and explained 29% of the variance in ΔPAS-liquid (R^2^ = 0.29; adjusted R^2^ = 0.21). Baseline PAS-liquid score was significantly associated with ΔPAS-liquid (B = −0.527, *p* = 0.001), with higher baseline PAS-liquid scores associated with a greater numerical reduction in PAS-liquid over time. Because the change score is mathematically coupled to its baseline value, this association was interpreted cautiously and was further examined using ANCOVA. In this analysis, using the post-treatment PAS-liquid score as the dependent variable and the baseline PAS-liquid score as a covariate, baseline 25-hydroxyvitamin D remained associated with the outcome (B = −0.021, *p* = 0.047; model R^2^ = 0.18), consistent with the change-score model. NIHSS was not an independent predictor in this model (B = −0.020, *p* = 0.617), whereas lower baseline 25-hydroxyvitamin D was associated with less improvement (B = −0.021, *p* = 0.047). Baseline 25-OH vitamin D and Δ25-OH vitamin D were examined. Longitudinal change in 25-hydroxyvitamin D (Δ25-OH vitamin D) was not independently associated with ΔPAS-liquid (B = −0.013, *p* = 0.145). Age, sex, and stroke localization were not significant predictors. Overall, these findings indicate that dysphagia rehabilitation was associated with significant improvement in oral intake and aspiration severity. However, all multivariable models were exploratory given the limited sample size and the risk of overfitting, and *p*-values were not adjusted for multiple comparisons; the observed biochemical associations should therefore be interpreted as hypothesis-generating.

## 4. Discussion

The principal findings of this study were outcome-specific. Neurological severity, as reflected by the NIHSS score, was independently associated with transition to oral feeding, whereas baseline PAS-liquid score was the strongest predictor of change in aspiration severity. In exploratory analyses, lower baseline 25-hydroxyvitamin D was additionally associated with a lower likelihood of transition to oral feeding and with less improvement in aspiration severity. The remaining systemic biochemical markers did not demonstrate consistent independent associations with the study outcomes. Given the limited sample size and the absence of correction for multiple comparisons, the biochemical findings should be regarded as exploratory and hypothesis-generating.

Additionally, patients who achieved oral feeding exhibited greater reductions in aspiration severity and a divergent change in vitamin B12 levels, suggesting that certain biochemical factors may have supportive or modulatory roles that require confirmation. Collectively, these findings indicate that improvement in oral-feeding status and aspiration severity is driven primarily by neurological impairment and baseline swallowing dysfunction, while nutritional status, particularly vitamin D, may play a complementary role that requires prospective confirmation.

In addition, significant improvements were observed in both swallowing-related outcomes and several systemic inflammatory markers following rehabilitation. The marked increase in FOIS scores and the significant reductions in PAS-soft and PAS-liquid scores indicate clinically meaningful improvements in oral intake and airway safety. Concurrent decreases in CRP and D-dimer levels, together with significant changes in WBC count, neutrophil count, lymphocyte percentage, and monocyte count, suggest that improvements in swallowing function may occur alongside favorable changes in the systemic inflammatory and hematological profile. These findings are consistent with previous studies suggesting that recovery from post-stroke dysphagia is not solely a function of local swallowing physiology but is also influenced by the patient’s overall biological condition [10,19].

The most important finding of our study is that the independent determinants of transition to oral feeding and improvement in aspiration severity were primarily clinical severity variables. In the main multivariable models, higher NIHSS score was associated with a lower likelihood of transition to oral feeding, whereas baseline PAS-liquid score was the strongest correlate of improvement in aspiration severity. These findings are in strong agreement with systematic reviews investigating recovery from post-stroke dysphagia, which have consistently identified stroke severity, instrumental evidence of airway compromise, and baseline dysphagia severity as the most robust clinical predictors of recovery [19]. In other words, the principal determinants of transition from enteral to oral feeding appear to be the initial neurological burden and baseline swallowing safety rather than the biochemical profile.

Nevertheless, our findings also suggest that the transition to oral feeding cannot be explained solely by neurological burden and baseline dysphagia severity and that systemic biological reserve may contribute to this process. The biochemical parameters included in the broad biomarker panel were evaluated individually in separate multivariable models adjusted for age, sex, NIHSS score, baseline PAS-liquid score, and stroke localization. Most systemic biochemical markers did not demonstrate consistent independent associations with the transition to oral feeding; however, baseline 25-hydroxyvitamin D showed an exploratory independent association in the Firth penalized model. Among the biomarkers investigated, albumin, vitamin B12, D-dimer, and 25-hydroxyvitamin D showed exploratory statistical patterns that warranted cautious secondary interpretation. These findings were not considered confirmatory and should be interpreted in the context of the multiple exploratory analyses performed.

Albumin is a commonly used marker of nutritional status and overall physiological reserve, and previous studies have associated malnutrition with poorer functional outcomes after stroke [3,10]. However, in the present study, albumin did not demonstrate a consistent independent association with transition to oral feeding after adjustment for clinical covariates. Therefore, the observed albumin findings should be considered exploratory and should not be interpreted as evidence of an independent prognostic effect.

The findings related to vitamin B12 can be interpreted within a similar neurometabolic framework. Vitamin B12 is an essential micronutrient for myelin integrity, neuronal transmission, and neurological recovery, and its deficiency has been associated with both ischemic stroke risk and adverse clinical outcomes [20,21]. Vitamin B12 showed a statistically significant between-group difference in change scores; however, the direction of change was heterogeneous, with a decrease among patients who transitioned to oral feeding and an increase among those who remained enterally fed. Because vitamin B12 did not demonstrate a consistent independent association in the multivariable analyses, this finding should be interpreted as exploratory and hypothesis-generating rather than as evidence of a favorable effect on feeding transition. However, because vitamin B12 was not subjected to detailed secondary analyses similar to those performed for vitamin D, these observations should be regarded as hypothesis-generating rather than confirmatory.

D-dimer reflects thrombotic and fibrinolytic activity, and elevated levels have previously been associated with adverse outcomes after ischemic stroke [7,8]. In the present study, however, D-dimer did not demonstrate a consistent independent association with transition to oral feeding after adjustment for clinical covariates. Therefore, its observed pattern should be interpreted as exploratory and as a potential marker of systemic biological burden rather than as an independent predictor of feeding transition.

Within this broader biological framework, 25-hydroxyvitamin D deserves particular attention. This is because vitamin D has increasingly been investigated not only as an indicator of general nutritional status but also as a potential biomarker related to post-stroke rehabilitation capacity, neuromuscular performance, and airway protection. The existing evidence remains inconsistent; while some studies and reviews have suggested beneficial effects of vitamin D supplementation on post-stroke rehabilitation outcomes, at least one randomized controlled trial failed to demonstrate a significant benefit [6]. Furthermore, the reported association between serum vitamin D levels and peak cough flow in patients with ischemic stroke suggests that this biomarker may be relevant not only to overall functional recovery but also to airway defense mechanisms associated with aspiration risk [22]. For these reasons, vitamin D was one of the biomarkers that warranted more detailed interpretation in our study.

In the exploratory multivariable analyses, lower baseline 25-hydroxyvitamin D levels were associated with a lower likelihood of transition to oral feeding and with less improvement in aspiration severity. However, these findings should be interpreted cautiously because of the limited sample size, model instability, and the absence of formal adjustment for multiple comparisons. Although baseline vitamin D demonstrated discriminatory ability in the ROC analysis, the identified cut-off value was clinically very low and should not be interpreted as a stand-alone clinical decision threshold. Accordingly, baseline vitamin D should be considered a candidate biological correlate rather than an established independent prognostic biomarker, and its potential role requires confirmation in adequately powered prospective studies. The present panel comprised non-specific systemic parameters; dysphagia-specific candidate biomarkers such as salivary substance P and calcitonin gene-related peptide were not assessed, and the conclusions should therefore be restricted to the investigated markers and to this retrospectively selected cohort.

### Limitations

This study has several limitations. In the absence of a control group, the observed changes cannot be attributed specifically to dysphagia therapy; in addition, baseline nutritional treatment and any prior dysphagia therapy were not consistently documented. In addition, the exact duration and nutritional composition of enteral feeding before baseline were not consistently documented; therefore, potential effects of nutritional treatment on the biochemical changes could not be fully separated from longitudinal clinical changes. Swallowing outcomes were assessed only with the Functional Oral Intake Scale and the Penetration–Aspiration Scale; measures of swallowing efficiency (e.g., pharyngeal residue, vallecular and pyriform sinus residue, bolus clearance, and standardized scales such as the MBSImP or DIGEST) were not available, so the study characterizes airway safety and feeding status rather than overall swallowing physiology. The videofluoroscopic protocol (bolus volumes and consistencies, number of swallows, rater number and expertise, blinding, and intra- and inter-rater reliability) and the content of the dysphagia rehabilitation were not standardized in this retrospective dataset, which limits reproducibility. Additional clinical variables that may influence dysphagia outcomes, including tracheostomy status, respiratory status, cough effectiveness, sarcopenia, detailed lesion location (brainstem versus supratentorial stroke), and previous dysphagia history, were not consistently available in the retrospective records and therefore could not be included in the analyses. The multivariable models were underpowered (approximately four events per variable), *p*-values were not corrected for multiple comparisons, and no external validation was performed, so the biomarker associations are exploratory. First, its retrospective single-center design limits causal inference and may restrict the generalizability of the findings. Second, the sample size may have constrained the stability of multivariable biomarker analyses, particularly for exploratory adjusted models. Third, because the main clinical models and the additional biomarker analyses were conducted at different analytical levels, the biomarker findings should be interpreted as complementary rather than definitive. Finally, although baseline vitamin D showed significant discriminatory performance in ROC analysis, the identified cut-off was clinically very low, which limits its direct applicability as a stand-alone decision threshold. Despite these limitations, the study has an important strength in evaluating post-stroke dysphagia not only through clinical swallowing measures but also together with inflammatory and nutritional biomarkers. This multidimensional approach provides a broader view of enteral-to-oral transition beyond swallowing safety alone.

## 5. Conclusions

Transition from enteral to oral feeding in patients with chronic post-stroke dysphagia was primarily associated with baseline neurological severity, particularly the NIHSS score. Improvement in aspiration severity was most strongly related to the baseline PAS-liquid score. Lower baseline 25-hydroxyvitamin D showed additional exploratory associations with both outcomes; however, these findings should be interpreted cautiously because of the retrospective design, limited sample size, and absence of correction for multiple comparisons. Overall, the findings emphasize the central role of clinical severity, while systemic biochemical markers may provide complementary hypothesis-generating information. These conclusions apply specifically to oral-feeding status and aspiration severity and should not be interpreted as representing overall swallowing recovery.

## Figures and Tables

**Table 1:** Demographic and clinical characteristics of the participants.

Variable	Category	n	%
Age (years)	Mean ± SD	57.5 ± 22.1	–
Time since stroke (weeks)	Mean ± SD	38.5 ± 7.8	–
Sex	Female	48	54
	Male	41	46
Oral feeding status	Transitioned to oral feeding	52	58
	Remained enterally fed	37	42
Stroke localization	MCA (Middle Cerebral Artery)	51	57
	ACA (Anterior Cerebral Artery)	20	23
	PCA (Posterior Cerebral Artery)	18	20

Abbreviations: SD, standard deviation; MCA, middle cerebral artery; ACA, anterior cerebral artery; PCA, posterior cerebral artery.

**Table 2:** Comparison of laboratory parameters and clinical scales before and after therapy.

Variable	n	Pre-Therapy (T0) Mean ± SD	Post-Therapy (T1) Mean ± SD	Z	*p*
CRP (mg/L)	89	51.76 ± 59.08	16.76 ± 34.18	−7.222	**<0.001**
Creatinine (mg/dL)	89	1.18 ± 1.08	1.53 ± 2.97	−0.447	0.655
Urea (mg/dL)	89	26.80 ± 13.67	27.65 ± 9.74	−1.403	0.161
ALT (U/L)	89	21.29 ± 10.85	22.45 ± 10.09	−1.012	0.312
AST (U/L)	89	22.57 ± 9.94	22.78 ± 7.51	−0.445	0.657
Sodium (mmol/L)	89	137.98 ± 4.48	139.25 ± 3.60	−2.099	**0.036**
Potassium (mmol/L)	89	4.15 ± 0.48	4.11 ± 0.55	−0.932	0.351
Vitamin B12 (pg/mL)	89	477 ± 195	459 ± 176	−0.843	0.399
25-OH Vitamin D (ng/mL)	89	17.85 ± 12.08	17.71 ± 13.78	−0.327	0.743
WBC (×10^9^/L)	89	9.46 ± 3.92	8.81 ± 4.62	−2.276	**0.023**
Hemoglobin (g/dL)	89	11.96 ± 2.65	12.49 ± 2.07	−1.452	0.147
Hematocrit (%)	89	36.83 ± 7.51	38.50 ± 6.16	−1.502	0.133
Platelet count (×10^9^/L)	89	263 ± 111	255.30 ± 84.69	−0.148	0.882
Procalcitonin (ng/mL)	89	0.63 ± 1.72	0.35 ± 0.63	−0.483	0.629
Neutrophil count (×10^9^/L)	89	6.68 ± 3.76	6.17 ± 6.69	−2.556	0.011
Neutrophil (%)	89	66.88 ± 16.49	65.44 ± 15.64	−0.945	0.344
Lymphocyte count (×10^9^/L)	89	1.84 ± 1.00	2.27 ± 3.63	−1.156	0.248
Lymphocyte (%)	89	19.87 ± 9.65	23.19 ± 11.12	−2.242	**0.025**
Monocyte count (×10^9^/L)	89	1.07 ± 3.08	0.68 ± 0.70	−2.310	**0.021**
Monocyte (%)	89	7.45 ± 2.69	7.06 ± 2.96	−1.451	0.147
Eosinophil count (×10^9^/L)	89	0.38 ± 1.16	0.20 ± 0.19	−0.516	0.606
Eosinophil (%)	89	2.36 ± 2.39	2.40 ± 2.27	−0.032	0.974
Basophil count (×10^9^/L)	89	0.10 ± 0.43	0.05 ± 0.05	−1.670	0.095
Basophil (%)	89	0.47 ± 0.55	0.52 ± 0.34	−2.182	**0.029**
Ferritin (ng/mL)	89	160.32 ± 320.37	98.48 ± 93.73	−1.211	0.226
D-Dimer (mg/L)	89	1.44 ± 0.80	0.99 ± 0.55	−7.624	<0.001
Albumin (g/dL)	89	4.05 ± 0.50	4.17 ± 0.49	−1.688	0.091
Total protein (g/dL)	89	6.91 ± 0.50	6.98 ± 0.57	−0.741	0.458
GGT (U/L)	89	20.63 ± 15.54	21.30 ± 27.75	−1.031	0.303
FOIS	89	1.87 ± 0.85	5.51 ± 0.97	−7.681	**<0.001**
PAS-Soft	89	5.80 ± 0.80	1.69 ± 0.71	−7.765	**<0.001**
PAS-Liquid	89	6.58 ± 0.74	2.03 ± 0.97	−7.713	**<0.001**

Comparisons between pre-therapy (T0) and post-therapy (T1) measurements were performed using the Wilcoxon signed-rank test. For some parameters, a small number of laboratory values were missing, so the effective sample size varies by parameter. Abbreviations: CRP, C-reactive protein; ALT, alanine aminotransferase; AST, aspartate aminotransferase; WBC, white blood cell count; GGT, gamma-glutamyl transferase; FOIS, Functional Oral Intake Scale; PAS, Penetration–Aspiration Scale; SD, standard deviation. Statistically significant *p* values are shown in bold (*p* < 0.05).

**Table 3:** Comparison of changes (Δ) in laboratory parameters, indices, and clinical outcomes between patients who achieved and did not achieve transition to oral feeding.

Δ Variable	Transitioned to Oral Feeding (n = 52) Mean ± SD	Remained Enterally Fed (n = 37) Mean ± SD	U	*p*
ΔCRP (mg/L)	−37.65 ± 51.12	−30.64 ± 38.32	612.0	0.832
ΔCreatinine (mg/dL)	0.26 ± 2.57	0.50 ± 3.72	681.0	0.748
ΔUrea (mg/dL)	−0.33 ± 18.20	2.80 ± 12.83	666.0	0.614
ΔALT (U/L)	−0.43 ± 12.87	3.86 ± 12.82	540.0	0.209
ΔAST (U/L)	−0.66 ± 11.56	1.64 ± 11.44	681.5	0.421
ΔSodium (mmol/L)	1.48 ± 3.92	0.91 ± 6.00	603.0	0.198
ΔPotassium (mmol/L)	−0.02 ± 0.75	−0.08 ± 0.75	646.0	0.358
ΔVitamin B12 (pg/mL)	−52.06 ± 275.41	36.74 ± 162.47	517.0	**0.049**
Δ25-OH Vitamin D (ng/mL)	0.20 ± 15.27	−0.71 ± 11.69	640.0	0.323
ΔWBC (×10^9^/L)	−0.76 ± 5.66	−0.48 ± 4.53	670.0	0.709
ΔHemoglobin (g/dL)	0.90 ± 2.36	−0.10 ± 1.79	557.0	0.071
ΔHematocrit (%)	2.50 ± 6.22	0.29 ± 5.71	565.0	0.093
ΔPlatelet count (×10^9^/L)	−5.46 ± 100.74	−12.14 ± 117.80	662.0	0.621
ΔProcalcitonin (ng/mL)	0.05 ± 0.60	−0.83 ± 2.86	620.0	0.240
ΔNeutrophil count (×10^9^/L)	−1.27 ± 4.73	0.77 ± 10.67	696.0	1.000
ΔLymphocyte count (×10^9^/L)	0.15 ± 0.74	0.89 ± 5.77	659.0	0.585
ΔMonocyte count (×10^9^/L)	−0.07 ± 0.25	−0.92 ± 5.16	665.0	0.686
ΔFerritin (ng/mL)	−47.36 ± 194.99	−85.81 ± 477.50	641.0	0.546
ΔD-Dimer (mg/L)	−0.42 ± 0.26	−0.50 ± 0.28	604.0	0.205
ΔAlbumin (g/dL)	0.11 ± 0.67	0.13 ± 0.33	672.0	0.666
ΔTotal protein (g/dL)	0.13 ± 0.74	−0.02 ± 0.53	630.0	0.344
ΔGGT (U/L)	−3.82 ± 16.38	8.09 ± 43.70	560.0	0.082
ΔFOIS	3.67 ± 1.67	3.59 ± 0.95	678.0	0.824
ΔPAS-Soft	−4.44 ± 0.68	−3.59 ± 0.91	214.0	**<0.001**
ΔPAS-Liquid	−4.90 ± 0.90	−4.00 ± 1.04	236.0	**<0.001**

Note: Δ values represent the change between post-therapy (T1) and pre-therapy (T0) measurements (Δ = T1 − T0). Note. Group comparisons were performed using the Mann–Whitney U test. Bold values indicate statistical significance (*p* < 0.05). The subgroup sizes shown are the group totals; for parameters with missing values, the effective group sizes are slightly smaller, and the whole-sample change reconciles with the subgroup values at these parameter-specific sample sizes. Abbreviations: CRP, C-reactive protein; ALT, alanine aminotransferase; AST, aspartate aminotransferase; WBC, white blood cell count; GGT, gamma-glutamyl transferase; FOIS, Functional Oral Intake Scale; PAS, Penetration–Aspiration Scale; SD, standard deviation.

**Table 4:** Firth penalized multivariable logistic regression model for transition to oral feeding.

Variable	B	SE	OR (Exp[B])	95% CI for OR	*p*
Baseline 25-OH Vitamin D (T0)	0.115	0.034	1.121	1.050–1.198	0.001
Δ25-OH Vitamin D (T1 − T0)	0.045	0.030	1.046	0.986–1.109	0.138
NIHSS score	−0.252	0.117	0.777	0.618–0.977	**0.031**
Age (years)	−0.005	0.021	0.995	0.955–1.037	0.828
Sex (male)	−0.796	0.619	0.451	0.134–1.517	0.198
Baseline PAS-Liquid score	0.073	0.425	1.076	0.468–2.475	**0.863**
ACA stroke (reference = MCA)	0.681	0.825	1.976	0.392–9.957	0.409
PCA stroke (reference = MCA)	0.903	1.845	2.467	0.066–91.789	0.625

Note: The dependent variable was transition to oral feeding (responder: 1 = yes, 0 = no). MCA was used as the reference category for stroke localization. Statistically significant associations are shown in bold. Abbreviations: OR, odds ratio; CI, confidence interval; NIHSS, National Institutes of Health Stroke Scale; PAS, Penetration–Aspiration Scale; MCA, middle cerebral artery; ACA, anterior cerebral artery; PCA, posterior cerebral artery.

**Table 5:** Independent predictors of improvement in aspiration severity: multivariable linear regression analysis.

Variable	B	SE	B (Std)	t	*p*	95% CI for B
Intercept	−0.030	1.187	–	−0.025	0.980	−2.399 to 2.340
Baseline 25-OH Vitamin D (T0)	−0.021	0.010	−0.245	−2.027	0.047	−0.042 to −0.000
Δ25-OH Vitamin D (T1 − T0)	−0.013	0.009	−0.179	−1.473	0.145	−0.032 to 0.005
NIHSS score	−0.020	0.039	−0.061	−0.502	**0.617**	−0.098 to 0.059
Age (years)	−0.006	0.008	−0.085	−0.767	0.446	−0.021 to 0.010
Sex (male)	0.013	0.224	0.006	0.057	0.954	−0.435 to 0.461
Baseline PAS-Liquid score	−0.527	0.155	−0.378	−3.397	**0.001**	−0.837 to −0.217
ACA stroke (reference = MCA)	−0.397	0.298	−0.162	−1.332	0.187	−0.992 to 0.198
PCA stroke (reference = MCA)	−0.581	0.457	−0.150	−1.271	0.208	−1.494 to 0.331

Note: The dependent variable was the change in aspiration severity measured by ΔPAS-Liquid (T1 − T0). MCA was used as the reference category for stroke localization. Statistically significant predictors are shown in bold. Abbreviations: NIHSS, National Institutes of Health Stroke Scale; PAS, Penetration–Aspiration Scale; MCA, middle cerebral artery; ACA, anterior cerebral artery; PCA, posterior cerebral artery; CI, confidence interval; SE, standard error.

## Data Availability

The data presented in this study are available on request from the corresponding author.

## References

[1] Martino R., Foley N., Bhogal S., Diamant N., Speechley M., Teasell R. (2005). Dysphagia after stroke: Incidence, diagnosis, and pulmonary complications. Stroke.

[2] Foley N.C., Martin R.E., Salter K.L., Teasell R.W. (2009). A review of the relationship between dysphagia and malnutrition following stroke. J. Rehabil. Med..

[3] Scrutinio D., Lanzillo B., Guida P., Passantino A., Spaccavento S., Battista P. (2020). Association Between Malnutrition and Outcomes in Patients With Severe Ischemic Stroke Undergoing Rehabilitation. Arch. Phys. Med. Rehabil..

[4] Nip W.F., Perry L., McLaren S., Mackenzie A. (2011). Dietary intake, nutritional status and rehabilitation outcomes of stroke patients in hospital. J. Hum. Nutr. Diet..

[5] Scalabrino G. (2009). The multi-faceted basis of vitamin B12 (cobalamin) neurotrophism in adult central nervous system: Lessons learned from its deficiency. Prog. Neurobiol..

[6] Borowicz W., Ptaszkowska L., Małecki R., Paprocka-Borowicz M. (2025). The Effect of Vitamin D Supplementation on Functional Outcomes in Patients Undergoing Rehabilitation After an Ischemic Stroke: A Prospective, Single-Blind, Randomized, Placebo-Controlled Study. J. Clin. Med..

[7] Yuan B., Yang T., Yan T., Cheng W., Bu X. (2021). Relationships Between D-Dimer Levels and Stroke Risk as Well as Adverse Clinical Outcomes After Acute Ischemic Stroke or Transient Ischemic Attack: A Systematic Review and Meta-Analysis. Front Neurol..

[8] Soldozy S., Yağmurlu K., Norat P., Elsarrag M., Costello J., Farzad F., Sokolowski J.D., Sharifi K.A., Elarjani T., Burks J. (2022). Biomarkers Predictive of Long-Term Outcome After Ischemic Stroke: A Meta-Analysis. World Neurosurg..

[9] Yoo J., Hong J.-H., Lee S.-J., Kim Y.-W., Hong J.M., Kim C.-H., Choi J.W., Kang D.-H., Kim Y.-S., Hwang Y.-H. (2020). Acute Kidney Injury after Endovascular Treatment in Patients with Acute Ischemic Stroke. J. Clin. Med..

[10] Chen H., Fu C., Fang W., Wang Z., Zhang D., Zhang H. (2025). Influence of nutritional status on rehabilitation efficacy of patients after stroke-a scoping review. Front Neurol..

[11] Ahmadabad M.A., Naeimi A., Keymoradzadeh A., Faghani S., Ahmadabad M.A., Boroujeni N.A., Mohammadpour H., Saber A. (2022). Evaluation of De Ritis (AST/ALT), ALP/ALT, and AST/ALP ratios as prognostic factors in patients with acute ischemic stroke. BMC Neurol..

[12] Tu W.J., Liu Q., Cao J.L., Zhao S.J., Zeng X.W., Deng A.J. (2017). Γ-Glutamyl Transferase as a Risk Factor for All-Cause or Cardiovascular Disease Mortality Among 5912 Ischemic Stroke. Stroke.

[13] Rosenbek J.C., Robbins J.A., Roecker E.B., Coyle J.L., Wood J.L. (1996). A penetration-aspiration scale. Dysphagia.

[14] Crary M.A., Mann G.D., Groher M.E. (2005). Initial psychometric assessment of a functional oral intake scale for dysphagia in stroke patients. Arch. Phys. Med. Rehabil..

[15] Kasner S.E. (2006). Clinical interpretation and use of stroke scales. Lancet Neurol..

[16] Firth D. (1993). Bias reduction of maximum likelihood estimates. Biometrika.

[17] Heinze G., Schemper M. (2002). A solution to the problem of separation in logistic regression. Stat. Med..

[18] Holick M.F., Binkley N.C., Bischoff-Ferrari H.A., Gordon C.M., Hanley D.A., Heaney R.P., Murad M.H., Weaver C.M. (2011). Evaluation, treatment, and prevention of vitamin D deficiency: An Endocrine Society clinical practice guideline. J. Clin. Endocrinol. Metab..

[19] D’Netto P., Rumbach A., Dunn K., Finch E. (2023). Clinical Predictors of Dysphagia Recovery After Stroke: A Systematic Review. Dysphagia.

[20] Zhou L., Song X., Wang J., Tan Y., Yang Q. (2023). Effects of vitamin B12 deficiency on risk and outcome of ischemic stroke. Clin. Biochem..

[21] Yahn G.B., Abato J.E., Jadavji N.M. (2021). Role of vitamin B12 deficiency in ischemic stroke risk and outcome. Neural Regen. Res..

[22] Yoo S.D., Park E.J. (2023). Serum vitamin D levels and peak cough flow in patients with subacute ischemic stroke. Medicine.

